# Practical Hints on Mounting

**Published:** 1881-12

**Authors:** R. N. Reynolds

**Affiliations:** D. & M. Junction, Detroit


### PRACTICAL HINTS ON MOUNTING.

BY R. N. REYNOLDS, D. & M. JUNCTION, DETROIT.

Standing before me is a dusty air-pump, a relic of the days in which we fought air bubbles in our mounts for the microscope. Well we remember those battles, how after the smoke of broken cover glasses and sticky fingers were cleared away, only a few bubbles were found and these had pluckily followed the object nearly out of the field.

Now the air pump has stopped never to go again in mounting, and instead of fighting the bubbles we keep clear of them.

We take our object (for balsam mounts) from spirits of turpentine and while well covered with the spirit place it on the slide and quickly apply the Canada balsam, before placing on the cover glass, and centre before it has time to settle, else the object will move with the cover.

For replacing objects found out of center we use a needle which has been driven eye first into a piece of soft wood for a handle, and then ground down on two opposite sides until only a thin blade of steel is left, this is to be warmed and inserted under the cover forcing the object back.

The mount freed from excess of balsam is to be kept in a warm place for about two days, then on the turn table the edge of the mount is given a couple of coats of shellac dissolved in alcohol, or one coat of Bell's cement to prevent the white zinc which is to follow from touching the balsam.

After drying another day replace it on the turn table and with pretty thick white zinc cement cover the shellac and edge of cover glass, and apply the color rings at once. For these we use French tube paints mixed in damar and thinned with benzole, thus with once centering on the turn table we have applied the zinc and as many rings as we wish. Allow a day or two for drying, next and last give the whole cement work a coat of best coach varnish which will preserve the glassy appearance of the work. It is important that artists' brushes of red sable be used. We use No. 8 for the white zinc, shellac, and varnish, and No. 1 for color rings.

If other readers of *The Microscope* will join me in giving their methods of mounting, etc., then I will give my methods of making, easily and quickly, neat hard cells of any desired depth for either opaque or transparent mounts, and many other points in mounting.

Let us help *The Microscope* and each other by showing up little bugs and the humbugs we have been induced to purchase to mount them with.

It would appear that nothing could be more simple than an examination of the contents of the mouth, but cases have occurred with us recently which go to change our belief on this subject; as a rule, however, it is an easy matter to remove some of the saliva to a slide, to wait a moment for the air-bubbles to rise when they can be broken or moved off with a needle, then to apply the cover and examine. But here one must be familiar with the different extraneous matters likely to be met with. Among them we might enumerate most of the starches, oil globules, portions of tissue, vegetable cells, the commercial fibres, the various "coatings" of the tongue, etc., and these mixed with sputa make a formidable mass.

